# Women’s experiences of social support during pregnancy: a qualitative systematic review

**DOI:** 10.1186/s12884-023-06089-0

**Published:** 2023-11-10

**Authors:** Mona Al-Mutawtah, Emma Campbell, Hans-Peter Kubis, Mihela Erjavec

**Affiliations:** 1 School of Human and Behavioural Sciences, Bangor University, Bangor, UK; 2 Community Medicine- Clinical Psychology, Kuwait University, Kuwait City, Kuwait

**Keywords:** Pregnancy, Social support, Systematic review, Qualitative research, Thematic synthesis

## Abstract

**Background:**

Social support during pregnancy can alleviate emotional and physical pressures, improving the well-being of mother and child. Understanding women's lived experiences and perceptions of social support during pregnancy is imperative to better support women. This systematic review explores and synthesises the qualitative research on women's experiences of social support during pregnancy.

**Methods:**

Databases PubMed, CINAHL, MEDLINE, APA PsycInfo and Scopus were searched with no year limit. Eligible studies included pregnant women or women who were up to one year postpartum and were assessed on their experiences of social support during pregnancy. The data were synthesised using the thematic synthesis approach.

**Results:**

Fourteen studies were included with data from 571 participating women across ten countries; two studies used focus groups, and 12 used interviews to collect their data. Four main themes were developed ('a variety of emotional support', 'tangible and intangible instrumental support', 'traditional rituals and spiritual support', and 'the all-encompassing natal home'), and six sub-themes ('female network connections', 'care and affection from the husband', 'dissatisfaction with relationships', 'financial support from the husband and family', 'practical support from family and friends', 'health information support').

**Conclusions:**

This systematic review sheds light on women’s experiences of social support during pregnancy. The results indicate a broad variety of emotional support experienced and valued by pregnant women from different sources. Additionally, women expressed satisfaction and dissatisfaction with tangible and intangible support forms. It was also highlighted that spirituality played an essential role in reducing stress and offering coping mechanisms for some, whereas spirituality increased stress levels for others.

**Supplementary Information:**

The online version contains supplementary material available at 10.1186/s12884-023-06089-0.

## Background

For some women, pregnancy is considered a time of joy, but it also involves many well-being, social, and physical changes (e.g., emotional, physiological, and relational changes). These changes during pregnancy can present many challenges [1–3]. For example, Yin et al. [4] conducted a systematic review to investigate the prevalence of antenatal depression during pregnancy across several continents. The results showed that the prevalence rates of any antenatal depression were 20.7%, and 15% of pregnant women experienced major antenatal depression, which is higher than general population 14.5% [5]. Other challenges reported in the existing literature are related to unplanned pregnancy, mood instability, physical health problems, financial problems, and a lack of social support during pregnancy [6–9]. For example, social support during pregnancy reportedly helps to alleviate the pressures of the pregnant women’s emotional and physical changes, and suggests to improve the mother and child’s well-being [10–12].

### The conceptualisation of social support

There is a wide range of literature connected to social support from many perspectives and disciplines over many decades of research [13–16]. Social support has broadly been outlined as a complex, multi-dimensional concept that can be defined as assistance provided by a person’s social network and involves the provision of emotional and physical support [16, 17]. However, from a traditional psychological perspective, Cohen and Wills [13] describe social support as support from social networks that can influence health through two pathways (direct effects and stress buffering). The direct-effect hypothesis suggests that social support can improve health regardless of whether the environment is stressful or not [18]. Further, it contributes to a sense of belonging and stability, resulting in improved self-esteem and reduced stress and mental health disorders [19]. Alternatively, the stress-buffering hypothesis posits that support may buffer against unhealthy reactions and provide the individual with access to additional resources that will enhance their capacity to cope with stressful events in two ways:

1. Perceived support can prevent a psychological or physiological stress reaction from arising when a potentially stressful event occurs. Consequently, perceived support may increase the perception that individuals can cope with negative events.
2. Perceived social support can intervene between the event of a stress reaction and the onset of a pathological process by reducing the stress reaction [19, 20].

### Social support during pregnancy

Kroelinger and Oths [21] explored the role of social support in wanted and unwanted pregnancies. The results indicated that unwanted pregnancies are strongly influenced by factors such as support from partners, the partner’s stability and status, and their feelings towards pregnancy. Therefore, Kroelinger and Oths highlights the potential role of a partner’s social support during pregnancy and shows how the lack of a partner’s support, particularly their emotional and practical support, can negatively affect women’s experiences by leading them to experience the pregnancy as unwanted. However, although, the relationship between a partner’s social support and whether a pregnancy is desirable seems simple, a person may decide that they want the pregnancy while it progresses based on certain discoveries, experiences, or events that are unrelated to the social support they receive from their partner. For example, parental social support can buffer the negative impacts of an unsupportive partner [22].

Likewise, Rini et al. [23] aimed to assess their experiences of the quality and quantity of social support they received from their partners, referred to as social support effectiveness (SSE). It focused on three functional types of social support: practical, emotional, and informational support. Greater SSE from partners predicted less anxiety during the second to third trimesters [23]. In addition, a recent systematic review of social support during pregnancy sought to investigate the relationship between social support and mental illness during pregnancy. A significant positive correlation between low social support and antenatal depression (14/15 papers), antenatal anxiety (6/8 papers), and self-harm (3/4 papers) was found [6]. Although these studies stressed that social support directly affects mental health, the pregnant women’s feelings, attitudes, perspectives, and past pregnancy experiences may mediate the relationship between a partner’s social support and the pregnant person’s anxiety [24]. This aligns with several studies that showed that those who perceive adequate social support during pregnancy are less likely to report stress, distress, or symptoms associated with anxiety and depression [25–27].

The above evidence demonstrates that social support may influence women’s experiences during pregnancy. However, more recent research has also incorporated contextual and situational factors associated with the COVID-19 pandemic. Since December 2019, the COVID-19 pandemic has affected almost all countries and territories and cases of COVID-19 increased exponentially worldwide [28]. Recent research by Meaney et al. [29] aimed to assess pregnant women’s perceptions and satisfaction with social support from an online survey conducted with 573 pregnant women during the pandemic from the US, Ireland, and the UK. The authors illustrated that a reduction in perceived social support that resulted from the lack of access to antenatal care during the COVID-19 pandemic increased negative feelings such as sadness, anxiety, and loneliness during pregnancy for these women. Although this kind of research can help healthcare providers determine strategies to help women during stressful times, further research is required to identify the types of social support (e.g., emotional, instrumental, etc.) that were most affected by the pandemic.

In conclusion, the existing literature affirms that social support during pregnancy plays a role in women’s well-being and physical health (amongst other areas). However, most of these studies primarily employed quantitative approaches [21, 23, 30–32]. This indicates that the existing studies would have been unable to capture any wider contextual factors which may also shape women’s experiences, the emotional aspects of social support, or experiential aspects of the topic [33]. Therefore, qualitative synthesis can provide an in-depth understanding of precious women’s experiences and perceptions of social support during pregnancy.

### Overall aim

This systematic review sought to analyse and synthesise all available qualitative evidence about women’s experiences of social support during pregnancy.

### Research question

According to Stern et al. [34] and Butler et al. [35], to formulate a good question, the four elements of the PICo (with a lowercase o) (as P referred to participants; I referred to phenomenon of interest; Co referred to context) mnemonic framework must be incorporated to identify the keywords to use in the review question. Table 1 outlines how the review research question was formulated using this framework.

**Table 1:** Critical appraisal skills programme (CASP) summary table

Authors and year of publication	Charvat et al. (2021) [36]			Chongo and Ngoma (2014) [37]			Clark (2001) [38]			Darvill et al. (2010) [39]			Eapen et al. (2019) [40]		
CASP Question	Yes	Can't tell	No	Yes	Can't tell	No	Yes	Can't tell	No	Yes	Can't tell	No	Yes	Can't tell	No
Was there a clear statement of the aims of the research?	•			•			•			•			•		
Is the qualitative methodology appropriate?	•			•			•			•			•		
Was the research design appropriate to address the aims of the research?	•					•	•			•			•		
Was the recruitment strategy appropriate to the aims of the research?	•			•			•			•			•		
Was the data collected in a way that addressed the research issue?	•				•		•			•			•		
Has the relationship between the researcher and participants been adequately considered?	•					•		•				•	•		
Have ethical issues been taken into consideration?	•			•			•			•			•		
Was the data analysis sufficiently rigorous?	•					•	•			•			•		
Is there a clear statement of findings?	•				•		•			•			•		
How valuable is the research?	•					•	•				•		•		
Scoring	10			5			9.5			8.5			10		

Authors and year of publication	Eddy and Fife (2021) [41]			Edmonds et al., (2011) [10]			Giblin et al. (1990) [42]			John-Akinola et al. (2022) [43]			Naz et al. (2021) [44]		
CASP Question	Yes	Can't tell	No	Yes	Can't tell	No	Yes	Can't tell	No	Yes	Can't tell	No	Yes	Can't tell	No
Was there a clear statement of the aims of the research?	•			•			•			•			•		
Is the qualitative methodology appropriate?	•			•			•			•			•		
Was the research design appropriate to address the aims of the research?	•			•					•			•	•		
Was the recruitment strategy appropriate to the aims of the research?		•		•				•			•				•
Was the data collected in a way that addressed the research issue?	•			•				•		•				•	
Has the relationship between the researcher and participants been adequately considered?	•				•				•			•	•		
Have ethical issues been taken into consideration?	•			•			•			•			•		
Was the data analysis sufficiently rigorous?		•		•			•					•	•		
Is there a clear statement of findings?	•			•					•	•			•		
How valuable is the research?	•			•			•			•			•		
Scoring	9			9.5			6			6.5			8.5		

Authors and year of publication	Puspitasari and Sulistyorini (2021) [45]			Raman et al. (2014) [46]			Reszel et al. (2014) [47]			Shakeri et al. (2021) [48]		
CASP Question	Yes	Can't tell	No	Yes	Can't tell	No	Yes	Can't tell	No	Yes	Can't tell	No
Was there a clear statement of the aims of the research?		•		•			•			•		
Is the qualitative methodology appropriate?		•		•			•			•		
Was the research design appropriate to address the aims of the research?		•		•			•				•	
Was the recruitment strategy appropriate to the aims of the research?		•		•			•			•		
Was the data collected in a way that addressed the research issue?	•			•			•			•		
Has the relationship between the researcher and participants been adequately considered?		•		•				•			•	
Have ethical issues been taken into consideration?	•			•			•			•		
Was the data analysis sufficiently rigorous?			•	•			•			•		
Is there a clear statement of findings?	•			•			•			•		
How valuable is the research?		•			•		•			•		
Scoring	6			9.5			9.5			9		

Therefore, the research question developed via the PICo framework [35] was: How do women experience the social support provided to them during pregnancy?

## Methods

### Search strategy

An extensive literature search was conducted using five electronic databases: PubMed, CINAHL Plus with full text, MEDLINE, APA PsycInfo, and Scopus between May 2022- February 2023. Shea et al. [49] notes that at least two databases should be searched for systematic reviews and meta-analyses; however, utilising more databases can yield more comprehensive and accurate results.

In addition, the Boolean connectors AND and OR were utilised to combine the following MeSH and search terms: “qualitative research”, “qualitative”, “qualitative method”, “interview”, “focus group”, “social support”, “psychosocial support systems”, “emotional support”, “family support”, “practical support”, “information support”, “pregnancy”, “pregnant”. Furthermore, following Butler et al. [35], a manual screening of the reference lists of all included studies was performed to identify additional potential studies.

In addition, the Enhancing the Transparency of the Reported Comprehensive Qualitative Research Statement (ENTREQ), was used to increase transparency (see Additional file 1) [50].

### Inclusion and exclusion criteria

The included studies met the following criteria:

i) Original qualitative studies published in English-language peer-reviewed journals with no year limit.
ii) The participants were mainly adult women over 18, although two papers included two pregnant women aged 17 in their samples.
iii) The participants were pregnant women or women who had given birth and were assessed on their experience of social support during pregnancy.
iv) The participants were pregnant women that were not specifically recruited because of pre-existing health issues or mental illness, as these conditions may affect their social support experiences.

The exclusion criteria:

i) Pregnant under age 17.
ii) Specific sub-groups of pregnant women (e.g., pregnant with HIV, diabetes, intellectual disabilities and visually impaired).
iii) Social support in breastfeeding.
iv) Unpublished and grey studies.
v) Theses and secondary research sources (e.g., reviews).

### Data extraction

As part of the review process, researchers can extract descriptive data (e.g., details of setting or context) and outcome data (e.g., results or conclusions) from the selected studies [51]. The critical information needed to extract the context and participants are the study setting, country, population, and participant characteristics. The information needed to describe the research design and methods is the methods for data collection, analysis, and findings [50, 51]. The information we included in Table 2 offers an overview of this data about the studies included in this review. A total of 14 international studies spanning 1990 to 2022 have been selected, from the United States (*n* = 5), Canada (*n* = 1), Bangladesh (*n* = 1), Indonesia (*n* = 1), Iran (*n* = 1), Pakistan (*n* = 1), India (*n* = 1), Zambia (*n* = 1), Nigeria (*n* = 1) and the UK (*n* = 1). In total, 571 adult women participated; two studies used focus groups and 12 used interviews to collect their data.

**Table 2:** Summary of included studies (*n* = 14)

Author(s), year, & country	Discipline	Aims/theoretical definition of social support	Sample & study population	Design	Methods	Findings
Giblin et al. (1990) [42], US	Health research	Aim: To address the construct of social support Definition: A multidimensional model of social support (instrumental, information and empathy and understanding) [52, 53]	300 women who were previously delivered, average age 23, two to five days postpartum. Asked about the construct of social support during pregnancy	Cross-sectional	Content analysis and structured interviews with open-ended and fixed-choice questions	Three social support factors emerged: - Intimacy - Comfort - Security
Clark (2001) [38], US	Medicine research	Aim: To explore the questions of who and what kinds of social support Mexicanas have during the perinatal period Definition: Support that is health-affirming in psychological and physical ways [54]	28 urban Mexican-origin women aged over 18 years in the last trimester	Longitudinal	Standard ethnographic methods and unstructured interviews	Four themes of social support: - Helping with daily hassles - Showing love and understanding - Being there for me - My family is failing me
Edmonds et al. (2011) [10], Bangladesh	Health research	Aim: To identify and characterise the functions of social support during pregnancy as women perceive it Definition: The function of one’s network [55, 56]	25 women aged 18–49 of multiple religions (23 Muslim, 2 Hindu) during the two months postpartum	Cross-sectional	A retrospective, cross-sectional design and in-depth semi-structured interviews	Four main types of support were mentioned: - Practical help with routines - Information - Emotional support and assurance - The provision of resources and material goods
Puspitasari and Sulistyorini (2021) [45], Indonesia	Nursing research	Aim: To identify the quality-of-life factors in pregnant women in East Java province Definition: not mentioned	13 pregnant women aged 20–39 years in the second or third trimesters Religion is not mentioned	Cross-sectional	Qualitative content analysis and semi-structured in-depth interviews	The social support factors of quality of life: - Husband’s support - Family support - Support of neighbours and friends - Sexual relations - Health service support
Charvat et al. (2021) [36], US	Psychology research	Aim: To explore how women who were pregnant during the Covid-19 pandemic communicatively made sense of their experience during pregnancy in light of their received social support Definition: Multiple types of social support (information, emotional, appraisal, and instrumental) [57–59]	21 women aged over 18 years, 10 of whom had given birth and 11 of whom were pregnant at the time of the interview Religion is not mentioned	Cross-sectional	Thematic analysis with an inductive and deductive approach and semi-structured interviews	Four themes related to social support emerged: - Connecting to give and receive emotional support to mitigate stress - Drawing on others’ knowledge for informational support - Receiving socially distant instrumental support - Lacking medical professional support
Reszel et al. (2014) [47], Canada	Health research	Aim: To explore the experiences of young pregnant and parenting women regarding behavioural expectations and behavioural changes during pregnancy, focusing on the individual and social context of their health behaviour experiences Definition: not mentioned	9 women with an average age of 19 who were pregnant or mothers of children under the age of 6 months Religion is not mentioned	Cross-sectional	Qualitative content analysis and semi-structured photo-elicitation interviews	The theme was: the influence of emotional support, with the subthemes: - A lack of emotional support and oppressive experience - Emotional support from partners and family offering empowering experiences
Raman et al. (2014) [46], India	Nursing research	Aim: To explore the wide-ranging sources of support that the infant dyad needs or expects throughout the perinatal period in urban India Definition: Psychosocial and cultural factors [46]	36 mothers aged 17–40 years who had experience pregnancy and childbirth within the last 2 years Religion is not mentioned	Cross-sectional	Ethnographic approach with in-depth qualitative semi-structured interviews	The themes: - The importance of women’s mothers - Women’s place or natal home - Female support network - The husband’s role - The variable role of the family
Shakeri et al. (2021) [48], Iran	Health research	Aim: To analyse the concept of social support for pregnant mothers Definition: A complex and multidimensional concept that refers to a person’s voluntary support for others, resulting in a positive response [60]	15 pregnant mothers aged over 18 years Religion is not mentioned Trimester is not mentioned	Cross-sectional	Qualitative phenomenological methods and in-depth semi-structured interviews	Three themes: - Information support (a lack of advice and counselling, lack of purposeful training courses, and the weakness of media education) - Need for satisfaction of medical and living needs - Spiritual support (religious and existential)
Naz et al. (2021) [44], Pakistan	Medicine research	Aim: To explore women’s perceptions regarding their husbands’ and in-laws’ support during pregnancy Definition: not mentioned	10 pregnant women in the third trimester Age and religion are not mentioned	Cross-sectional	Qualitative exploratory design, thematic analysis, and in-depth semi-structured interviews	Three themes: - Lack of comprehensive support mechanisms for emotional, physical, psychological, housekeeping, and financial support - Physical and mental strain (fatigue, natural mood swings, violence, and abuse) - Barriers to maternal services (through socio-economic status and inappropriate behaviour from husbands and in-laws)
John-Akinola et al. (2022) [43], Nigeria	Psychology research	Aim: To investigate women’s perspective on the social support that men provide during pregnancy (What support do pregnant women expect from their partners? What experiences do women have with partner support during pregnancy? How can men support their pregnant partners during pregnancy?) and factors that could influence or promote this support (What factors prevent men from fully supporting their partners?) Definition: not mentioned	41 women (mean age 36.3 + 4.90 years) with babies between 0 and 6 months Religion is not mentioned	Cross-sectional	Thematic analysis and focus group discussions	- Expected support: psychological and sexual satisfaction support - Way to provide support: Helping with house chores and taking care of other children - Women’s experiences with men supporting their loss of appetite and irritation suggest that men should be tolerant to provide support, encouragement, and patience during pregnancy - Factors that influence men’s support: a bad attitude towards the husband and nagging about a lack of support
Eapen et al. (2019) [40], US	Psychology research	Aim: To explore the perceptions and experiences of social support during pregnancy among low-income women who had recently given birth to an LBW infant Definition: not mentioned	15 women aged 18 years or older who had given birth within the past 9 months Religion is not mentioned	Cross-sectional	Qualitative descriptive design and in-depth semi-structured interviews	Three themes emerged: - Women’s experience of pregnancy - Challenges faced by women during pregnancy - Availability of essential support (emotional, physical, and informational) and the father and female relatives as major sources of support
Chongo and Ngoma (2014) [37], Zambia	Nursing research	Aim: To explore and describe pregnant women’s perceptions of husbands’ provision of support during pregnancy and labour to encourage men’s participation in maternal health services to improve maternal health Definition: not mentioned	34 pregnant women aged 20 to 49 years Religion and stage of pregnancy are not mentioned	Cross-sectional	Descriptive exploratory design and content analysis and focus group discussions	Support during pregnancy with household chores and preparing for the baby: - Need emotional support from the husband - The husband should give financial support to provide for basic needs, transportation and supplies for the new baby - The husband should accompany the pregnant person to the health facility during labour
Darvill et al. (2010) [39], UK	Midwife research	Aim: To explore the maternal transition from women’s perspectives and identify any unmet support needs Definition: not mentioned	13 women with an average age of 31 who had delivered within 6 to 15 weeks Religion is not mentioned	Cross-sectional	Grounded theory, constant comparative methods, and semi-structured interviews	The supporting theme: - Mothers, partners, and peers offered support - Mothers provided emotional, practical, and informational support - The practical support from mothers was related to helping with shopping, housework, or cooking - There was a lack of emotional support from the husband
Eddy and Fife (2021) [41], US	Psychology research	Aim: To build a theory describing how husband’s involvement during pregnancy impacts the couple relationship	11 married couples (husbands aged 25–36 years, wives aged 24–34 years), 7 nonreligious and 15 religious, who had had a baby within 2 to 6 months	Cross-sectional	Grounded theory and semi-structured interviews	Developed a theory of active husband involvement, consisting of four components: - Helping with a positive attitude - Instrumental support - Emotional support - Strong couple relationship postpartum

*USA* United States of America**,** *COVID-19* coronavirus disease 2019, *LBW* low birthweight, *UK* United Kingdom

### Quality appraisal

A quality assessment of the studies included was conducted using the Critical Appraisal Skills Programme for Qualitative Studies Checklist (CASP). The CASP tool is endorsed by both Cochrane and the World Health Organization for the qualitative synthesis of evidence [61]. According to Butler et al. [35], the scoring system for the answers to each question was Yes = 1, Can’t tell = 0.5, and No = 0 points; high-quality papers earned 9–10 points, moderate-quality papers, 7.5–9, and low-quality papers, less than 7.5. The first author (MA) was the first appraiser and a second independent reviewer (MP) evaluated five randomised studies to verify the robustness of the process [62]. Next, the results were compared, and the reviewers’ assessments were found to be similar across the sample studies. Table 2 summarises the results of the critical appraisal.

### Data and thematic synthesis

Thematic synthesis, developed by Thomas and Harden [63], was used to generate new insights from the primary studies. This approach consists of three stages: coding the text, developing descriptive themes, and generating analytical themes. This method provides an explicit process for reducing qualitative data by utilising different reporting styles, such as thin descriptions and multiple quotations [62, 64]. Each article’s results section was stored on a Word file and manually analysed using free line-by-line analysis. Then, these free codes were organised into related areas to construct descriptive themes, and, ultimately, these were developed into analytical themes [65]. The coding process was conducted inductively, and all of the preliminary codes and the descriptive and analytical themes were discussed and refined by the independent reviewer (EC), between July and September 2022. The themes were also further discussed under supervision for expert supervisory input, review, and iterative development (ME, PH, & EC). This process supported the quality of the theme generation. After this iterative process, four main themes and six sub-themes were developed from 126 codes.

## Results

### Search outcome

**Fig. 1:**
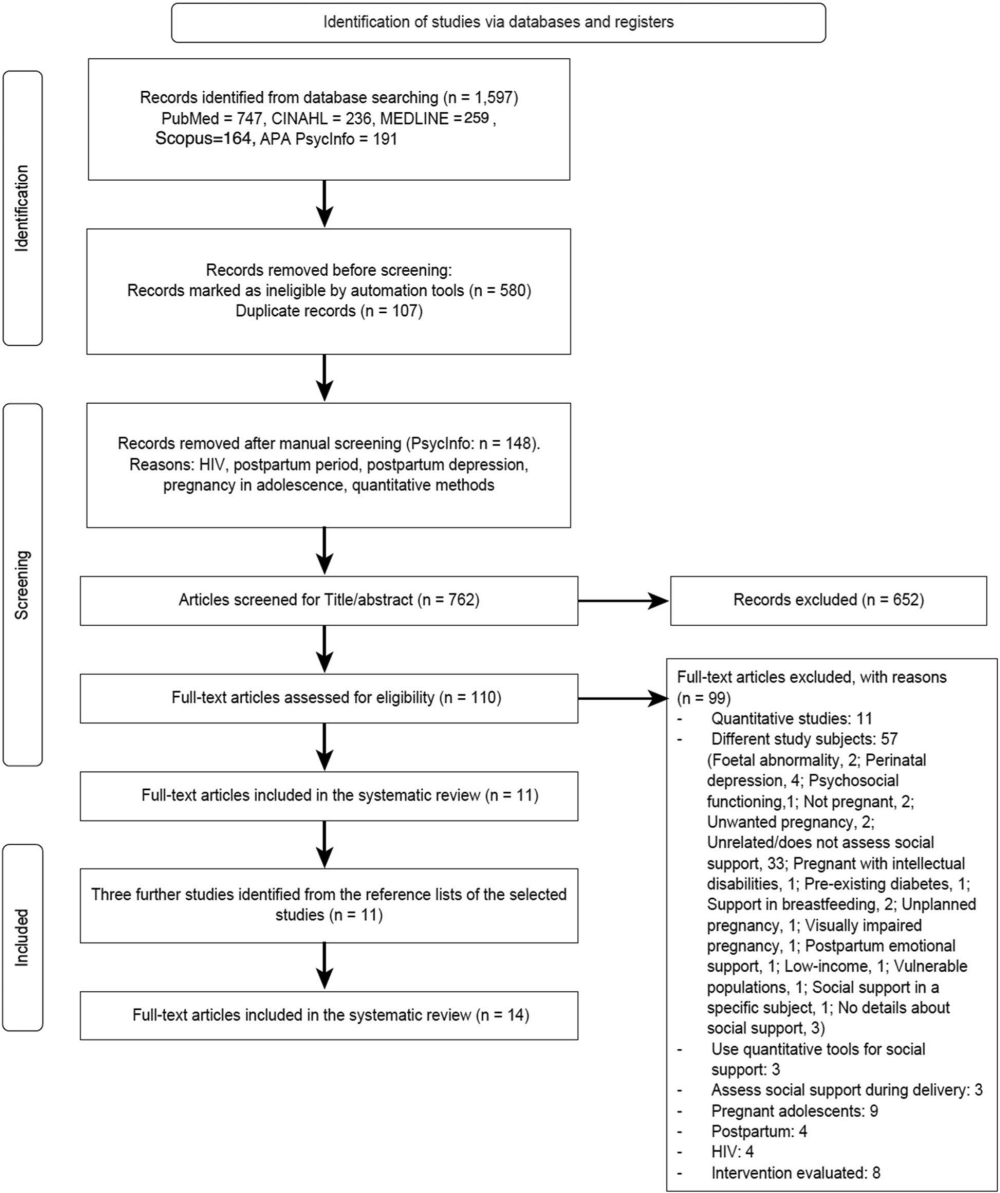
PRISMA diagram outlining the search process

A total of 1,597 articles were identified by the initial search. After 107 duplicates and 148 unsuitable studies were removed, 762 titles and abstracts were screened and a further 652 were excluded. The full text of 110 articles was retrieved and screened by the first author for eligibility and 99 articles did not meet the inclusion criteria. After reviewing the reference lists of the remaining studies, two additional studies that met the inclusion criteria were identified. One final study was found while writing the first draft that met the inclusion criteria. Thus, a total of 14 qualitative studies were reviewed with the research teams, who decided to include all 14 in this systematic review. The PRISMA Flow Diagram shows a detailed description of the study selection process (Fig. 1).

### Results of the thematic synthesis

**Fig. 2:**
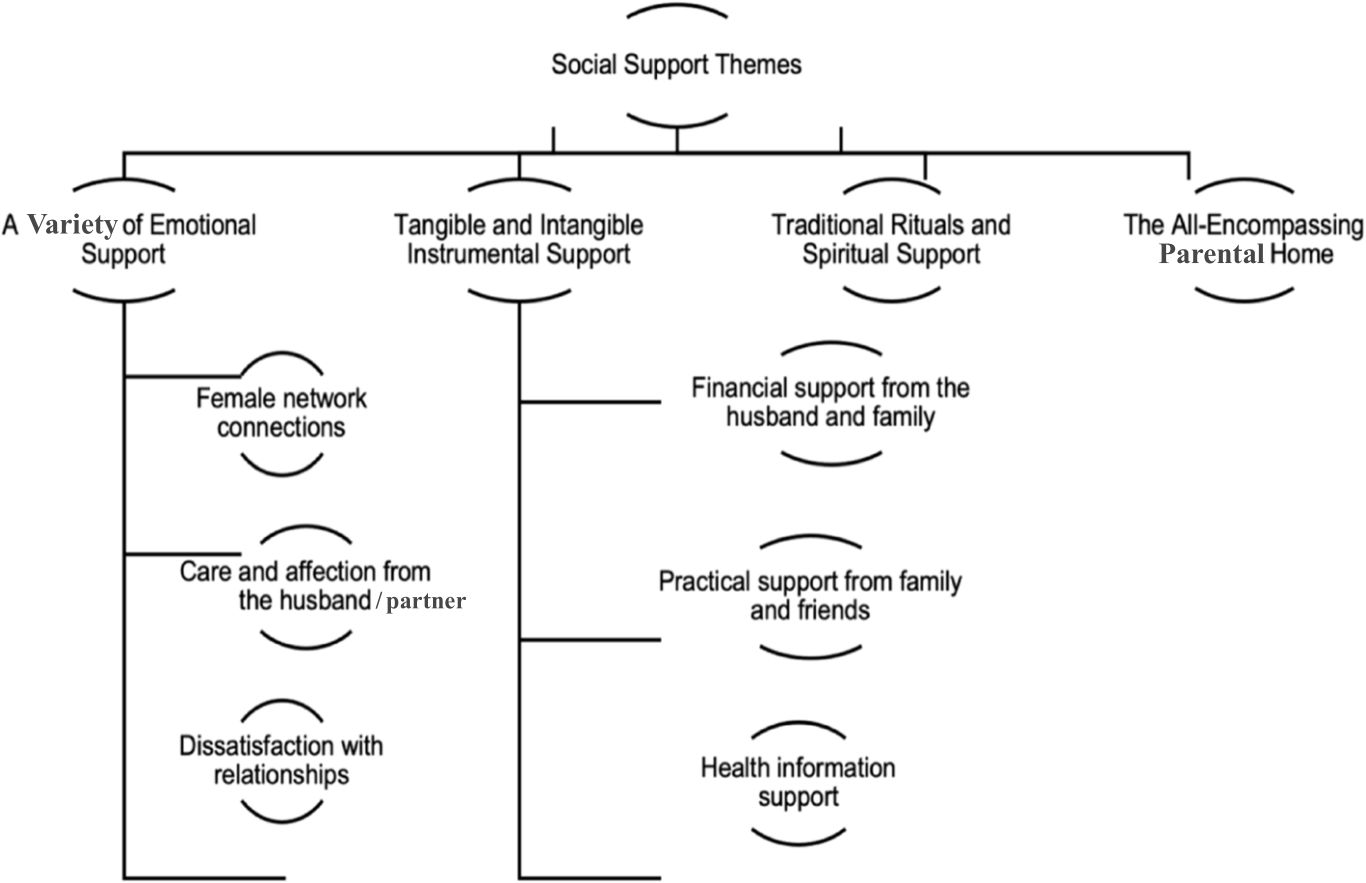
Analytical themes

The four main themes (see Fig. 2) generated through the thematic synthesis are “a variety of emotional support”, “tangible and intangible instrumental support”, “traditional rituals and spiritual support”, and “the all-encompassing natal home”. Each of the main themes and sub-themes is discussed below in more detail.

#### A variety of emotional support

This main theme describes women’s experiences of emotional support during pregnancy. The data from the included papers illustrated that the participants experienced increased emotional support during pregnancy from their female networks and marital relationships [10, 36, 38, 40, 42–48]. For some, the experience of emotional support existed across a variety, including some women who reported dissatisfaction.

##### Female network connections

This sub-theme offers insight into a range of sources from which the women had previously gained emotional support during pregnancy. The participants highlighted that they gained emotional support through their connections with other females within their social networks [10, 36, 38–40, 46]. Female emotional support could be afforded by family members (e.g., mothers, grandmothers, sisters, or sisters-in-law) or other female friends and neighbours. Pregnant women described emotional support as the act of women expressing care, sharing, and expressing feelings and supportive words, and listening to them. Some women also referenced how their mothers offered emotional support during the perinatal period and how they perceived this support as essential throughout their pregnancy.

*“There are enough people around me to talk to and support, (but) mainly I would tell my mother about everything. She has been very supportive throughout” *[46]*.*

*“Sister-in-law told me: ‘Do not get afraid, nothing will happen,’ when I felt the pain, she told me, ‘Do not worry, nothing will happen’” *[10]*.*

Moreover, some women mentioned that during the COVID-19 pandemic, when social restrictions were in place, receiving gifts from loved ones expressed love and care between the female relatives and friends and the pregnant women.

*“Due to social restrictions, Kelly’s mother and sisters sent a stroller and a car seat to celebrate her pregnancy” *[36]*.*

In addition to receiving gifts during Covid-19, some women revealed that connecting via social media with both pregnant and non-pregnant friends helped them to alleviate their stress and ensured that they were not alone during this challenging time.

*“Cause like my husband was great support too, but to communicate with someone who is more in your shoes is helpful” *[36]*.*

The above shows how the women’s connections with others offered a variety of emotional support both before and during the Covid-19 pandemic.

##### Care and affection from the husband/ partner

This sub-theme outlines a range of emotional support that the women experienced from their husband or partner during pregnancy. This was sometimes described as how their husband’s paid attention to them, encouraged them with supportive words, and allowed them space to discuss their concerns of the pregnancy [36, 37, 40–42, 44, 45, 47].

*“I feel lucky that I have somebody that’s willing to let me go take a bath and not be consumed in playing a video game or something. He was always listening to what I needed”* [41]*.*

In addition, this shows that some husbands met women’s needs when they were patient during pregnancy, avoiding any conflict or arguments [43]. Meanwhile, husbands prevented their pregnant wives from having to do hard manual labour, such as working in a factory, as an expression of affection and care [45]. Care can also be exemplified through the husband taking care of their wives’ diets.

*“My husband is so loving and caring; he takes care for my diet, he brings me ½ kg milk and fruits on daily basis”* [44]*.*

##### Dissatisfaction with relationships

This sub-theme gives insights into the other end of the spectrum, showing how some women experienced a lack of emotional support during pregnancy (highlighted in 6 of the 14 studies included) [38–40, 44, 47, 48]. For example, some women discussed how their husbands or partners were less caring and did not focus on their health or the health of their babies. There was also dissatisfaction when their husbands did not understand any emotional changes that they may have experienced.

*“My husband does not ask me what the doctor said about me and baby’s condition; when I come back from the doctor’s clinic, he is careless”* [44]*.*

Furthermore, women without close family and friends or who live far from them described themselves as lonely or helpless [38, 39, 44, 47].

*“I know almost no one here. I met a woman, but she moved, and now there’s no one”* [38]*.*

#### Tangible and intangible instrumental support

This main theme illustrates a range of instrumental social support that the women experienced during pregnancy. As part of this theme, tangible support refers to material aids, e.g., the provision of money or goods and behavioural acts, such as helping with household chores [18, 66]. Alternatively, intangible support describes directive guidance, such as information, advice, constructive feedback, and affirmation about the women’s health during pregnancy [67]. The first and second subthemes, “financial support from the husband and family” and “practical support from family and friends” relate to tangible support, and the third, “health information support”, to intangible support.

##### Financial support from the husband and family

This sub-theme describes how some of the participants appeared to be satisfied with their financial situation and the financial support they received from their husbands or families. However, this was not true of all women; some were dissatisfied with the financial support available to them. The source of financial support varied but included the participants’ husbands and parents and grandparents [10, 37, 40, 42, 44, 48]. One of the participants expressed how her husband’s financial support uplifts her mood.

*“I want him to buy baby items for the baby or he gives me money to buy. This makes me feel good because it shows that he is concerned about my situation”* [37]*.*

##### Practical support from family and friends

For this sub-theme, the women’s husbands and in-laws were referred to as sources of practical help. Some women also mentioned their mothers and friends, although the participants rarely asked them for practical support [10, 36–39, 43–46]. Forms of practical support included helping with household chores, cooking, childcare, shopping, and taking the pregnant person to their antenatal appointments. Women perceived their husbands as providers of many aspects of practical support during pregnancy, particularly assisting with daily housework, taking them to natal clinics, and providing childcare for their children [10, 37, 45, 46]. These tasks were also mentioned in connection to other family members, such as the women’s mothers, and friends.

*“My husband used to help me with things I could not do. For example, carrying water for cows” *[10]*.*

*“When I was vomiting for the first few months, three different friends used to cook different dishes for me every day; they looked after me so well” *[46].

Furthermore, some participants highlighted how their family and friends provided practical support during the COVID-19 pandemic. They talked about how their loved ones helped them with grocery shop and run errands.

*“They’ll call and be like, ‘Do you need me to get you anything? I’m at the store, that way you don’t have to go out”* [36].

##### Health information support

This sub-theme provides insight into how healthcare professionals were considered sources of information and advice, in addition to people such as mothers, friends, and pregnancy group members. This included positive feedback to normalise the pregnant person’s experience, information about the foetus’s health condition, nutrition advice and information about delivery [10, 36, 38–40, 45, 46, 48].

Some participants never mentioned healthcare providers as routine sources of information support but occasionally referred to them when describing acute problems related to pregnancy, the health of the foetus, or delivery information [40, 45, 46]. Other women, especially mothers, were routine sources of information and advice.

*“My mother had 11 children, out of which seven survive… therefore she gave all advice (during pregnancy). And I followed her advice”* [46]*.*

During the Covid-19 pandemic, some pregnant women faced a lack of informational support from healthcare providers about the Covid-19 virus and lockdown regulations. This led them to rely upon their pregnancy group peers to discuss concerns online through social media. For example, Charvat et al. [36] referred to the following quotes by two participants:

*“They don’t really tell me [anything]. And [my obstetrician] makes me feel comfortable as he tells me not to worry. He says I’m not high-risk, etc. But no, my OB doesn’t talk much about the actual virus.”*

These examples give an overview of the different forms and sources of health information support in the context of the Covid-19 pandemic and beyond.

#### Traditional rituals and spiritual support

This main theme refers to any culturally specific support (i.e., to avoid certain types of foods or exercises and to go out at noon) or any cultural or traditional practices during pregnancy that centre the well-being of the mother and child [10, 36, 38, 40, 45, 48]. The examples presented below will demonstrate how the women reported various forms of such support, including advice on movement and exercise, traditional foods, and avoiding evil spirits [10, 38]. These forms of support usually came from grandmothers, mothers, sisters-in-law, and aunts, as some participants mentioned in Edmonds’s et al. [10] study:

*“You cannot go out at noon, evening, dawn, and night. Evil spirits will catch you.”*

*“She (sister-in-law) forbade me to move in a clumsy way. She told me to be careful about movement and timing of movement.”*

Spiritual support, in the context of this review, refers to relying on the ‘higher spiritual being’ (as some participants called it God and others called it Allah) to protect and reduce stress and pain during pregnancy via prayers from the pregnant women and their husbands, mothers, and grandmothers. The concept of spiritual support signifies the religious dimension of a relationship with a preferred power (e.g., God, Allah, or Brahman) [48]. All pregnant women in the selected studies highlighted God as a form of spiritual support, mentioning trusting God, praying to God to reduce stress, and faith in God’s protection [10, 36, 38, 40, 45, 48].

*“Prayer and supplication to God help me calm down and reduce my stress”* [48]*.*

Spiritual support was not only experienced by the pregnant women but was a shared relational experience among them, their husbands, and other close family members. During the Covid-19 pandemic, faith in God was a type of support that one participant mentioned.

*“When asked how she feels about the information she has received from her doctors, Becka said, ‘Confused. Nothing set in stone… Yeah, the information I get is all over the place. And so, I mean, the best I can do is keep myself protected and hope to God that it keeps me protected’”* [36]*.*

Moreover, some participants reported religious group support during their pregnancy, mainly through counselling and financial support. The religious group support gave these women a sense of security and gave them the strength to persevere through this difficult time. It also gave them a sense of purpose, knowing that they had a support system, and that God was in control.

#### The all-encompassing parental home

The final main theme refers to how the participants expressed their experiences of social support within their family homes. For example, the parental home is the home of the pregnant woman’s parents, which usually, as the participants revealed, was the setting in which all their needs were met during pregnancy. This theme was developed from three studies assessing social support in Pakistan, India, and Bangladesh [10, 44, 46]. Most of the women from these countries mentioned their parental home as an integral part of their lives and described their parental home as a place where they feel happy and relaxed and receive more care.

*“My parents thought if I will be there [in-laws’ house], I will have to work, so I won’t be able to take rest also, that is why they [my parents] brought me here”* [46]*.*

The review affirmed that during pregnancy, women tend to spend more time in their parental homes, as their families often look after them in terms of chores, cooking, and cleaning [10].

## Discussion

This review sought to analyse and synthesise all available qualitative evidence about women’s experiences of social support during pregnancy. The participants in the included studies described a broad variety of social support experiences, including emotional, instrumental, and informational support. Four main themes were generated: “a variety of emotional support”, “tangible and intangible instrumental support”, “traditional rituals and spiritual support”, and “the all-encompassing parental home”.

This review found that pregnant people received emotional support from a diverse range of people within their social networks, gaining helpful support in terms of coping mechanisms and the regulation of stress during pregnancy and the COVID pandemic [10, 36, 42, 43, 45, 46]. These findings align with Rini et al. [23], who found that emotional and intimate support from the marital relationship and the husband supports adjustment during pregnancy and increases well-being. Kolker et al. [68] also found that emotional support was particularly critical during the pandemic, as many women experienced isolation and loneliness. The lack of physical connection with family and friends due to the pandemic caused many women to experience higher levels of stress, anxiety, and feelings of loneliness. Women’s emotions are regulated through social support and their relationships, which, in turn, may reduce emotional exhaustion during pregnancy due to the fear of childbirth or fears of having a child born with illness [69, 70]. The importance of emotional support (e.g., listening and affectionate interactions) is evident and consistent with the findings of this review [10, 36, 41].

Conversely, this review also highlighted a lack of or dissatisfaction with the emotional support that some of the participants experienced [38–40, 44, 47, 48]. Insufficient family support harmed pregnant women’s maternal behaviour and health. For example, Fernandez and Newby [71] used interviews to explore the extent to which pregnant women of Mexican descent in the United States were supported by their families and partners. Their results indicated an association between family support and the circumstances of the pregnancy. Women without cohabiting relationships with the baby’s father before becoming pregnant received less emotional support from their families, particularly their mothers. These women were, therefore, less likely to look forward to prenatal care, adopt a healthy behaviour (e.g., smoking), or be excited about giving birth to their babies.

This finding also highlights the importance of instrumental support, such as financial, informational, and practical support during pregnancy. As it revealed that a lack of financial support increased stress and dissatisfaction, while the provision of this support increased feelings of safety, emotional support, and being cared for and not alone [10, 40, 42, 44, 48]. Therefore, although instrumental (e.g., financial, or informational) support may be seen as merely practical support, these types of behaviours may make the woman feel that she is loved, cared for, and supported: that she matters [37]. Thus, the practical aspects of social support may not be considered separate categories of support but occasionally interacting forms of social support.

Dissatisfaction with the husband’s or partner’s practical support also was found as part of the thematic synthesis [43, 44]. However, when considering the role of practical support, a gap in the literature appears regarding many cultural factors. For instance, many studies argue that culture should be considered when trying to understand perceived satisfaction or dissatisfaction with the support given [72–74]. For example, in Western cultures, fathers’ roles have evolved from being only breadwinners to partners who play an active role in all aspects of their children’s lives [41]. However, women in Middle Eastern cultures (Saudi Arabia and Iran) are assumed to be primarily responsible for traditionally feminine tasks and homemaking, including attending to children’s needs, cleaning, and cooking, whereas men are the key breadwinners and are responsible for traditionally masculine tasks, such as making money, and home repairs [75, 76]. This is an important argument as cross-cultural differences or the impact of culture on pregnant women’s perceptions were not considered in most of the studies included in this review, which might be one of its critical limitations.

The included studies highlighted how important informational support was for women to understand or make sense of their health-related experiences and gather information during pregnancy. This included positive feedback to normalise their experience, information about the foetus’s health condition and nutrition advice. Gist-Mackey et al. [77] suggested that informational support has previously been determined vital during times of uncertainty and stress (such as pregnancy and COVID-19 pandemic), supporting other literature affirming that informational support can decrease stress, anxiety, and ambiguity among pregnant women [78, 79].

The theme of traditional rituals and spiritual support was also generated as part of the thematic synthesis. This was considered to take several different forms, such as prayers and advice on traditional food and avoiding evil spirits [10, 36, 38, 40, 45, 48]. The role of traditional cultural customs, values, and beliefs has been explored in the existing literature. For instance, Ayaz and Efe [80] described how some people in Turkey believe that, if the pregnant woman eats quince during pregnancy, the baby will be born with dimples. However, the overall results of this review indicated that some pregnant women perceived traditional rituals as a way to show concern and care [10, 36, 38]. Prayer was a form of spiritual support that the participants in the included studies relied on during pregnancy to reduce stress and seek protection [10, 36, 38, 45]. However, the role of spirituality and varying spiritual beliefs regarding the experience of stress may also be culturally influenced and shaped. In the current literature, the stress-buffering impact of spirituality may be higher among people from more conservative, religious cultures (e.g., Iranians), compared with those who are less religious, less conservative cultures. For example, a study conducted in Iran found that spirituality can reduce stress, particularly stress related to pregnancy [81]. It is also important to acknowledge that the role of spiritual support may have not only positive effects; as Mann et al. [82] emphasise, spirituality was associated with increased perceived stress among pregnant Hispanic people living in the US. The most likely explanation lies in the phenomenon of reverse causation (women with higher levels of stress seek comfort in religion). Therefore, the impact of spirituality on perceived stress may also be affected by the culture, among other broad factors (i.e., social, cognitive, personal, emotional, situational, or demographic factors).

During pregnancy, the parental home appeared important, with visits to this home described as motivated by the desire to reduce the pregnant person’s workload [10, 44, 46]. The benefits of visiting the parental home were often related to the women being surrounded by their families, who offered social and practical support. The finding that pregnant women experienced yearning for their parental homes can be explained by the theory of the collectivism/ individualism dichotomy as this theme arose from three studies performed in collectivist cultures (India, Bangladesh, and Pakistan). The theory of the collectivism/individualism dichotomy argues that individualistic cultures prioritise the needs of individuals over the needs of the group as a whole. Collectivist cultures are characterised by strong emotional bonds and social relationships between society members, especially mothers, whereas individualistic cultures have weaker emotional bonds [76, 83]. Individuals from more collectivistic backgrounds reported feeling less alone and experiencing more social support from their families than those from individualistic backgrounds [84, 85]. Therefore, further studies on individualistic cultures are needed to confirm these findings. However, they are consistent, from a broad psychological perspective, with the stress-buffering model, which emphasises that social support may buffer stress and contribute to a sense of belonging and stability, resulting in improved self-esteem and reduced stress [28, 86–88].

### Strengths and limitations

This review is the first one to consider qualitative research on women’s experiences of social support during pregnancy, which may inform future research designs. Additionally, it examines women's experiences of social support from all over the world and takes a rigorous and systematic approach. It reveals how social support and other factors impact women's well-being during pregnancy and what types of support they value. This review also provides insight into pregnant experiences during COVID-19 and provides some preliminary findings derived from recent research. However, the failure to conduct a sufficiently exhaustive search for studies is a potential limitation of any review, including this one [89]. Also, the small number of studies in this review curtail the richness and depth of the analysis of individual subjective meanings, making the findings difficult to generalise. Although the generalisation of qualitative findings is not typically the aim of qualitative research, generalisation does allow the analysis to be transferred to other contexts and settings [33]. Moreover, most of the included papers were from low-income countries; thus, future studies are needed to examine women’s experiences in wealthy countries.

### Implications

Healthcare providers (e.g., nurses, psychologists, or social workers) should raise awareness about the importance of providing pregnant women with the required social support (e.g., emotional, instrumental, and informational) through their social circles, including the husband, mother, father, and female networks. Healthcare providers should tailor social support interventions to meet individualised needs as women’s needs may differ. For example, Dennis et al. [90] found that women who participated in breastfeeding peer support interventions valued emotional support most and were less focused on education and informational support, although many social support interventions focus on informational and educational aspects [91]. Interventions are most effective when they are developed based on the needs of the target population [92]. It is therefore important for healthcare providers to not adopt a one-size-fits-all approach, but rather tailor their services and interventions in order to meet the specific and diverse needs of women within their communities, based on research and data analysis results. Moreover, healthcare providers need to screen pregnant women to know what they value in receiving support and assess their level of emotional and practical support during pregnancy. This screening should be followed by encouraging the inclusion of the key support people (friends, family members, and partners) throughout pregnancy [43, 47]. Yawn et al. [93] concluded that 654 of 1,897 women had elevated screening scores indicative of depression. This is significant as it shows that many women needed additional help and resources for mental health issues. Mental health screenings can provide valuable information to help identify those needing extra support. Thus, by integrating formal mental health screening into a wider assessment and taking into account the factors highlighted in this study, health professionals could achieve a more person-centred, holistic, and effective provision.

Additionally, policymakers and other relevant stakeholders should consider a community-based social support program for pregnant women as a means of helping them cope with the challenges of pregnancy. By providing such programs, we can facilitate a more comprehensive approach to maternal care, acknowledging that emotional and social support is an essential component of the well-being of a pregnant woman.

## Conclusions

This systematic review provides insight into women’s experiences of social support during pregnancy. The results indicate that pregnant women experienced and valued a wide variety of emotional support from different sources, including their female networks, husbands, families, and parents. Furthermore, women experienced a mixture of tangible and intangible support and reported satisfaction and dissatisfaction with these kinds of support. In addition, the review highlighted the role of spirituality and how this was sometimes perceived as reducing stress and offering a coping mechanism, whilst for others, spirituality increased the stress experience. Overall, the results of this review provide insight into a range of experiences associated with social support in pregnancy.

### Supplementary Information

**Additional file 1. **Enhancing the transparency in reporting the synthesis of qualitative research: the ENTREQ statement.

## Data Availability

The datasets used and/or analysed during the current study are available. from the corresponding author on reasonable request.

## Abbreviations

SSE: Social support effectiveness
COVID-19: Coronavirus disease 2019
UK: United Kingdom
USA: United States of America
ENTREQ: Enhancing the Transparency of the Reported Comprehensive Qualitative Research Statement
MeSH: Medical Subject Headings
PRISMA: Preferred Reporting Items for Systematic Reviews and Meta-Analyses
CASP: Critical Analysis Skills Programme
